# Comparative analysis of white matter signal alterations in dementia with Lewy bodies and Alzheimer's disease: a systematic review and meta-analysis

**DOI:** 10.3389/fradi.2025.1554345

**Published:** 2025-04-04

**Authors:** Asad Abdi, Milad Alipour, Milad Ghanikolahloo, Amin Magsudy, Fatemeh HojjatiPour, Ali Gholamrezanezhad, Mehran Ilaghi, Mehran Anjomrooz, Fatemeh Sayehmiri, Ramtin Hajibeygi, Mobina Fathi, Reza Assadsangabi

**Affiliations:** ^1^ Neurologist Specialist, Department of Neurology, MIRDIF Hospital Dubai, Dubai, United Arab Emirates; ^2^ Neurologist Specialist, Department of Neurology, Iranian Hospital Dubai, Dubai, United Arab Emirates; ^3^ Department of Medicine, Islamic Azad University Tehran Medical Sciences, Tehran, Iran; ^4^ King's College Hospital NHS Foundation Trust, London, United Kingdom; ^5^ Islamic Azad University Tabriz Branch Faculty of Medicine, Tabriz, Iran; ^6^ Student Research Committee, School of Medicine, Shahid Beheshti University of Medical Sciences, Tehran, Iran; ^7^ Keck School of Medicine of University of Southern California, Los Angeles, CA, United States; ^8^ Department of Radiology, Cedars Sinai Hospital, Los Angeles, CA, United States; ^9^ Institute of Neuropharmacology, Kerman Neuroscience Research Center, Kerman University of Medical Sciences, Kerman, Iran; ^10^ Department of Radiology, Advanced Diagnostic and Interventional Radiology Research Center, Medical Imaging Center, Imam Khomeini Hospital Complex, Tehran University of Medical Sciences, Tehran, Iran; ^11^ Skull Base Research Center, Loghman Hakim Hospital, Shahid Beheshti University of Medical Sciences, Tehran, Iran; ^12^ School of Medicine, Tehran University of Medical Science, Tehran, Iran; ^13^ Department of Radiology and Biomedical Imaging, Yale School of Medicine, CT, United States

**Keywords:** Alzheimer's disease, dementia with Lewy bodies, white matter hyperintensities, meta-analysis, dementia

## Abstract

**Background and aim:**

Lewy body diseases (LBD) include neurodegenerative diseases such as Parkinson's disease (PD), dementia with Lewy bodies (DLB), and Parkinson's disease dementia (PDD). Because DLB and Alzheimer's disease (AD) share similar neurological symptoms, DLB is frequently underdiagnosed. White Matter Hyperintensities (WMH) are associated with dementia risk and changes in both DLB and AD. In order to examine WMH discrepancies in DLB and AD patients and gain insight into their diagnostic utility and pathophysiological significance, this systematic review and meta-analysis is conducted.

**Material and methods:**

Databases such as PubMed, Scopus, Google Scholar, and Web of Science were searched for studies reporting WMH in DLB and AD patients based on Preferred Reporting Items for Systematic Review (PRISMA) guideline. Stata version 15 US is used to analyze the extracted data.

**Results:**

Twelve studies with 906 AD and 499 DLB patients were considered in this analysis. Although not statistically significant, the WMH was 0.03 ml larger in AD patients than in DLB patients. The prevalence of hypertension varied, ranging from 21% to 56% in DLB patients and from 30% to 52% in AD patients. Different findings were found on the prevalence of diabetes; some research suggested that DLB patients had greater rates (18.7%–37%) than AD patients (9%–17.5%). The imaging modalities FLAIR, T2-weighted, and T1-weighted sequences were employed. Compared to DLB patients, AD patients had higher cortical and infratentorial infarcts.

**Conclusion:**

Those with AD have greater WMH volumes than cases with DLB, suggesting that WMH can be a biomarker to help better differentiation between these neurodegenerative diseases; however, this difference is not significant. To better understand the therapeutic implications and options for reducing WMH-related cognitive loss in various patient populations, more research is necessary.

## Introduction

Lewy body disorders (LBD) are characterized by the accumulation of aggregated *α*-synuclein protein in Lewy bodies and Lewy neurites. LBD encompasses three main disorders: dementia with Lewy bodies (DLB), Parkinson's disease (PD), and Parkinson's disease with dementia (PDD) (1).

DLB is often underdiagnosed due to its symptom overlap with Alzheimer's disease (AD) and PDD, with only about 30% of cases correctly identified. Symptoms of DLB include cognitive decline, rapid eye movement (REM), sleep behavior disorder, executive dysfunction, and various motor impairments such as bradykinesia and gait disturbances (2).

The most common differential diagnosis of DLB is AD. The hallmark of AD, a neurodegenerative condition marked by amnestic dementia, impaired memory, language, visuospatial function, and executive function, often accompanied by neuropsychiatric symptoms (3). The clinical similarity between DLB and AD complicates their distinction, as both can cause dementia (4). This overlap is partly due to shared pathophysiological features, including amyloid-beta and tau aggregation in DLB, akin to AD, which is the main pathologic mechanism of AD (5).

Clinical differentiation can hinge on specific symptoms including, AD patients typically exhibit early and prominent cognitive decline due to encoding errors, while DLB patients show more retrieval-related cognitive impairments, along with parkinsonism and complex visual hallucinations uncommon in advanced AD (2, 6). Despite these distinctions, clinical differences are often subtle, necessitating additional assessments, such as imaging. Various imaging techniques, such as structural and functional magnetic resonance imaging (MRI), have been effective in differentiating DLB from AD (7, 8).

Additionally, nuclear imaging with different radiotracers has been highlighted as a valuable tool for diagnostic and differential diagnosis purposes (9). On the other hand, according to the statement, Flupane single-photon emission computed tomography. (FP-CIT SPECT) or Metaiodobenzylguanidine (MIBG) are useful modalities to differentiate between these diseases in their advanced stages, and they appear to favor DLB (4).

Two reliable techniques for differential diagnosis have been identified by prior research; (18) F-fluorodeoxyglucose (FDG)-PET and (123) I-iodoamphetamine (IMP)-SPECT (10).

In one study that was conducted by Miyagawa et al. (11), it is stated that ^123^I-FP-CIT SPECT using specific software analysis of the putamen is a very good discriminative imaging modality in this matter. It is also understood by Colloby et al. (12) that a combination study with electroencephalography (EEG) biomarkers and MRI visual rating scores have a greater diagnostic accuracy than individual modalities. Meanwhile, the slowing of the dominant EEG rhythm is a sensitive and specific modality by itself, according to Bousiges et al. (4) study.

Last but not least are the changes in the white matter. White matter hyperintensities (WMH), commonly seen in T2-weighted images, are indicative of vascular risk factors in the elderly and are associated with an increased risk of dementia, AD, and vascular dementia. Studies have mentioned that there are various ways that WMH, which causes multiple pathological changes, can cause dementia,

WMH is more prevalent in AD and DLB than in PDD, making it a potential diagnostic marker (5, 13, 14). Therefore, it can be used as an indicator of AD and DLB. While numerous systematic reviews have examined WMH in one specific neurodegenerative disorder, few have compared disorders to evaluate the overlapping pathophysiology. Assessing WMH aspects like location, volume, and pathophysiology in both AD and DLB can enhance understanding of WMH's role in cognitive decline, aiding researchers and clinicians alike. Subsequently, tremendous effort has been made up to this point to establish a precise diagnostic tool for individuals with dementia. We aim to outline studies on the use of WMH as a diagnostic tool and estimate the diagnostic value of these changes in the brains of AD and DLB patients.

## Materials and method

This systematic review and meta-analysis follows the recommendations set forth by Preferred Reporting Items for Systematic Review (PRISMA) (15).

### Search strategy

The present systematic review and meta-analysis aims to assess WMH changes in DLB and AD patients compared to the control group. This study was conducted by reviewing the original documents published until March 2024. We used databases including PubMed, Web of Science, Scopus, Google Scholar, and EMBASE. The keywords “white matter hyperintensities” were combined with either “Lewy body disease” or “Alzheimer's disease”, and the search was limited to English language documents.

### Inclusion and exclusion criteria

Our criteria for inclusion of studies in this systematic review and meta-analysis was reporting the volume of WMH in DLB and AD patients compared to a control group. In order to identify and choose the relevant topics, two independent researchers first evaluated the articles by their titles and abstracts, removing duplicate documentations. The whole text of the chosen papers was then evaluated independently by two authors. Studies with inadequate data, not reporting WMH, not specifying AD or DLB were excluded. A total of 12 qualitative research studies were taken into consideration for this analysis; however, studies without appropriate data and non-human studies were removed.

### Quality assessment

To evaluate the quality of the included research, we employed the Newcastle-Ottawa Quality Assessment Scale (NOS) (16). The eight criteria on this scale examine and evaluate the caliber of pertinent studies. Based on the Ottawa checklist for cross-sectional studies, the components evaluate choice, comparability, and outcome. Studies can be categorized into four categories based on their final NOS checklist scores: very good quality (9–10 score), good quality (7–8 score), satisfactory quality (5–6 score), and unsatisfactory quality (0–4 score) (Table 1).

**Table 1:** Characteristics of included studies.

Author (year)	Country	*n* patients DIB	*n* controls	Mean age patient DLB (SD)	Mean age control (SD)	Sex, male patients DLB (%)	Sex, male controls (%)	WMH score patient DLB (SD)	WMH score controls (SD)	WMH score region DLB patients	*n* hypertension in patients (%) DLB(OR)	*n* hypertension in controls (%)	*n* diabetes mellitus in patients (%) DLB (OR)	*n* diabetes mellitus in controls (%)	AD *n*	AD patients Age (SD)	Sex, male patients AD (%)	WMH score patients AD (SD)	WMH score region AD patients	*n* hypertension in patients (%) AD	*n* diabetes mellitus in patients (%) AD	Imaging sequences	NOS assessment score
Baik et al. 2023 (29)	Korea	19	37	75.4 (8.8)	8.8	7 (36)	12 (32)	1.5 (0.6)	1.1 (0.3)	DWMH = 1.5 ± 0.6 mm PWMH = 1.8 ± 0.8 mm					22	69.8 (9.1)	8 (36)	1.6 (0.6)	DWMH = 1.6 ± 0.6 mm PWMH = 1.5 ± 0.7 mm			T1w	8
Saeed Mirza et al. 2022 (20)	Canada	44		64.6 (10.1)		28 (63)		3 (3.9)		DWMH =0.5 ± 0.8 cm^3^ PWMH = 2.5 ± 3.4 cm^3^	14 (31.8)		2 (4.5)		202	72.1 (9.5)	91 (45)	6.9 (9.7)	DWMH = 0.8 ± 1.0 cm^3^ PWMH = 6.0 ± 9.3 cm^3^	71 (35.1)	21 (10.4)	T1w T2w	7
Dadar et al. 2022 (26)	Canada	26		72.49 (8.02)		23 (88%)		17.59 (13.52)		Frontal = 7.00 ± 4.72 cm^3^ Parietal = 5.74 ± 5.73 cm^3^ Temporal = 2.11 ± 2.37 cm^3^ Occipital = 2.70 ± 1.93 cm^3^					88	74.54 (7.77)	51 (58%)	13.81 (9.58)	Frontal=6.13 ± 5.10 cm^3^ Parietal=3.97 ± 3.75 cm^3^ Temporal=1.69 ± 0.93 cm^3^ Occipital=2.01 ± 1.15 cm^3^			T1w FLAIR	7
Park et al. 2021 (44)	Korea	45	34	73.1 (7.7)	71.9 (6.3)	15 (33.3)	14 (41.2)	1.29 (0.55)	1.18 (0.58)	DWMH=1.29 ± 0.55 mm PWMH=1.49 ± 0.63 mm	27 (60)	11 (32.4)	17 (37.8)	6 (17.6)	57	73.5 (7)	23 (40.4)	1.35 (0.67)	DWMH=1.35 ± 0.67 mm PWMH=1.42 ± 0.60 mm	29 (50.9)	10 (17.5)	T1w T2w	8
Caso et al. 2021 (22)	Italy	24	20	72 (5.7)	69.6 (6.7)	17 (70)	9 (45)	4.6 (4.8)	0.5 (0.5)	Total Frontal Parietal Temporal Occipital	13 (54.2)		4 (16.7)		26	71.1 (5)	9 (34)	1.7 (1.5)	Total Frontal Parietal Temporal Occipital	10 (41.7)	6 (25)	T1w T2w	9
Baik et al. 2021 (28)	Korea	19	37	75.4 (8.8)	70.2 (5.6)	8 (36)	12 (32)	1.8 (0.8)	1.1 (0.3)	PWMH=1.8 ± 0.8 mm DWMH = 1.5 ± 0.6 mm					22	69.8 (9.1)	8 (36)	1.5 (0.7)	PWMH=1.5 ± 0.7 mm DWMH=1.6 ± 0.6 mm			A Philips 3.0 MR scanner	9
Joki et al. 2017 (24)	Japan	50	50	76.4 (5.6)	75.6 (6.5)	30 (60)	28 (56)	1.74 (0.63(	1.16 (0.58(	DSWMH=1.74 ± 0.63 PVH = 1.92 ± 0.70	28 (56)	19 (38)	8 (16)	9 (18)	50	76.3 (6.7)	26 (52)	1.70)0.61(	DSWMH=1.70 ± 0.61 PVH=1.92 ± 0.72	27 (54)	10 (20)	T2w	8
Sarro et al. 2016 (21)	USA	81	240	72 (8)		67 (82)		25.3 (21.1)	15.6 (15.7)	Deep, periventricular, and subcortical lobes	35 (44)		7 (9)		240	75 (10)	135 (56)	24.3 (21.9)	deep, periventricular, and subcortical lobes	123 (52)	16 (7)	T1w T2w FLAIR	9
Mendes et al. 2015 (23)	France	91		70.9 (9.81)		38 (41)		1.5 (0.92)		Total	35 (38.5)		17 (18.7)		67	73.9 (8.64)	28 (41)	1.7 (0.93)	Total	19 (28.4)	9 (13.4)	T1w T2w FLAIR	9
Fukui et al. 2013 (19)	Japan	59		78.1 (6)		25 (42%)		1.05 (0.71)		Total	29 (49%)		18 (31%)		81	79.5 (5.4)	38 (46)	1 (0.77)	Total	48 (59%)	26 (32%)	T2w FLAIR	8
Burton et al. 2006 (18)	UK	14	33	74.2 (8.1)	74.4 (6.3)	8 (57)	19 (57)	5 (1.875)	5 (2.806)	PVH(percent brain volume) = 0.3 (0.2–0.7) ml DWMH (percent brain volume) = 0.05 (0.01–0.17) ml	3 (21%)	8 (24%)	3 (21%)	2 (6%)	23	78.2 (4.6)	13 (56)	14 (9.183)	PVH (percent brain volume) = 1.0 (0.4–3.0) ml DWMH (percent brain volume) = 0.24 (0.06–0.94) ml	7 (30)	2 (9)	FLAIR	8
Barber et al. 1999 (27)	UK	27	26	75.9 (7)	76.2 (5)	19 (70)	14 (53)	23 (3)	19 (1)	Frontal=22 ± 2 mm Temporal=3 ± 0 mm Parietal=15 ± 1 mm Occipital=2 ± 0 mm					28	77.4 (5)	10 (35)	25 (4)	Frontal=25 ± 2 mm Temporal=4 ± 0 mm Parietal=13 ± 0 mm Occipital=3 ± 0 mm			T2w	7

### Data extraction

Two separate authors independently screened and assessed all included studies via NOS, and the third author resolved any discrepancies. The following data were extracted for final analysis: sample size, mean age of patients, sex, male patients, amount of WMH and prevalence rate of hypertension and diabetes in DLB and AD patients.

### Statistical analysis

To screen for differences in white matter volume in patients with DLB and AD, a meta-analysis was executed using Stata version 15 US. Data extraction and meta-analysis were carried out, provided that each study had enough data. In this study, the prevalence rate was chosen as the unit of measurement for the effect size. Standardized mean difference (SMD) between DLB and the AD patients was analyzed according to the prevalence of WMH in these patients. Following the guidelines of the random effects model, data was evaluated. I^2^ statistics were used to evaluate heterogeneity and values higher than 50% were flagged for higher heterogeneity. With the use of Funnel plots and Begg and Egger's regression test, publication bias was objectively measured.

### Publication bias

**Figure 1:**
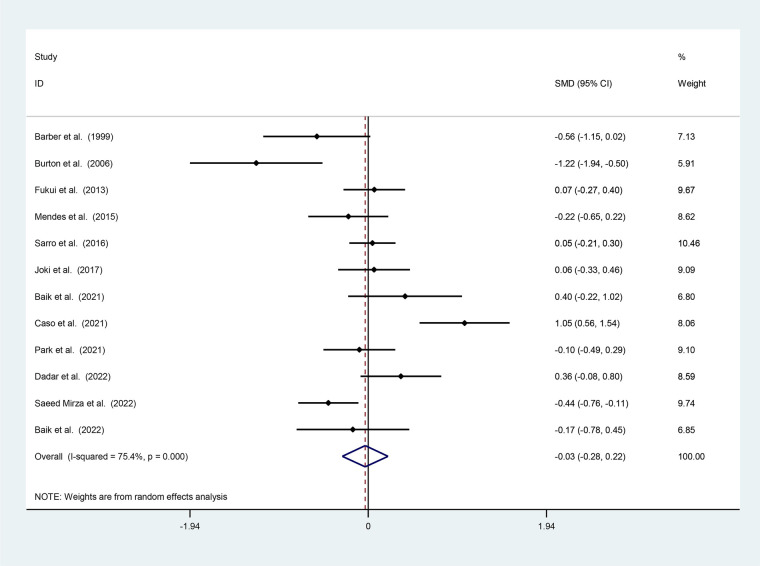
Funnel plot of the publication bias.

**Figure 2:**
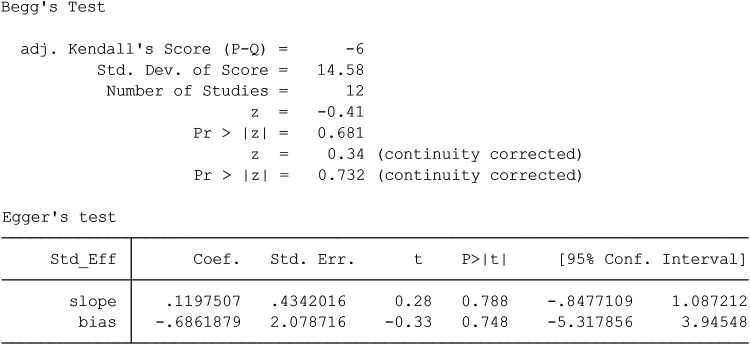
PRISMA flow diagram.

To evaluate publication bias in the selected literature, Egger and Begg's test (17) was used; Following the rules, *P* < 0.05 indicates a significant publishing bias (Figures 1, 2). Further, it was done using a linear regression analysis with intercept and slope parameters. The formula yi ¼ a + bxi+ɛi [i = 1… r (r = the number of studies), yi = standardized estimate, xi = precision of studies, ɛi = error terms] was used to compute the named parameters.

## Results

### Study selection and characteristics

**Figure 3:**
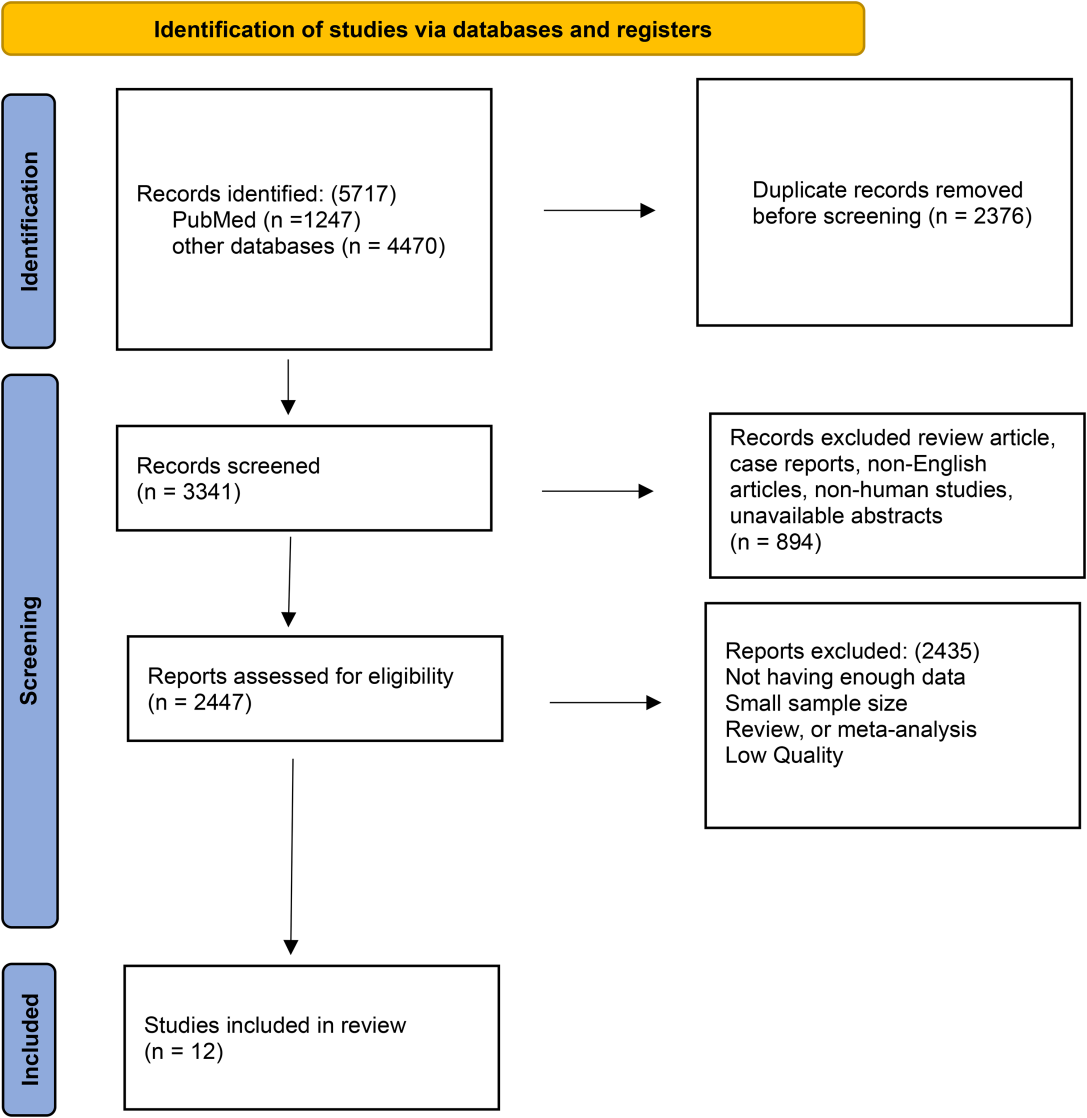
Begg and egger's publication bias.

Based on the PRISMA criteria, the current study was conducted (15). Out of the 5,727 papers found in our initial search, 2,376 duplicate papers were excluded. The 3,351 remaining records were examined. Then, 894 articles were removed during title and abstract screening and also because of low quality, unclear or limited information, or both. 12 studies were eligible after full-text screening of the remaining publications and were included in the systematic review (Figure 3). In the present study, 499 DLB patients were compared with 906 AD patients and 477 control group patients. The participants’ ages ranged from 64.6 to 78.1 years (Table 1).

### WMH variations in DLB and AD patients

**Figure 4:**
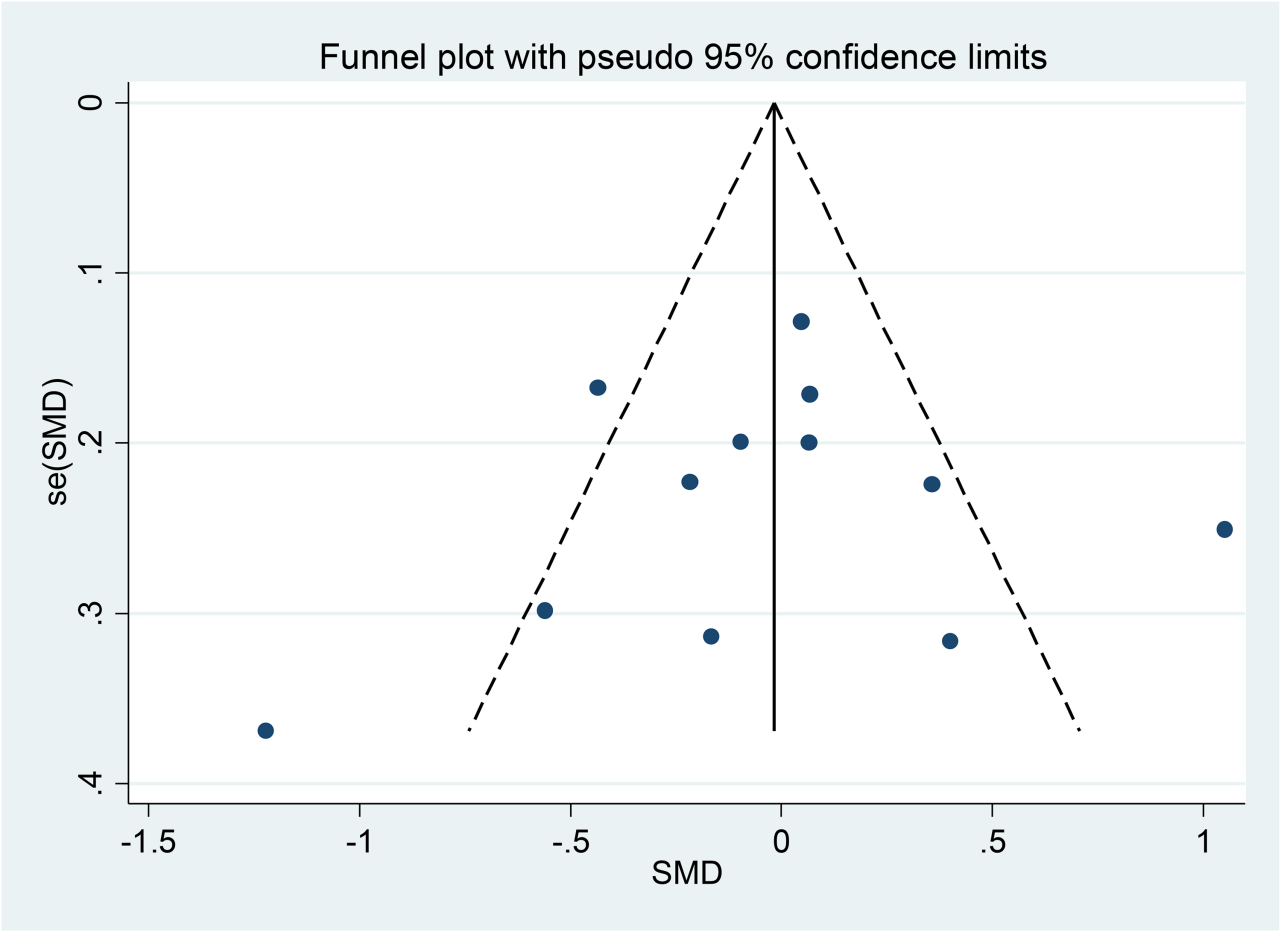
Forest plot of the standard mean difference (SMD) of WMH between patients with LBD and AD. The weight of each paper on the meta-analysis is indicated by each parallelogram, the 95% CI is visualized by the interval within the boundaries. Literature is presented based on random effect model.

Twelve studies were selected based on the volume of WMH in patients with DLB and AD (18–29). The analysis included 499 DLB cases with a mean age of 73.37 ± 7.80 years and 906 AD cases with a mean age of 74.26 ± 7.31 years. Our results show that the WMH volume is 0.03 ml (95% CI: −0.28–0.22; I2 = 75.4%) higher in AD patients compared to DLB patients (Figure 4), though this difference is not statistically significant.

### The prevalence of hypertension in DLB patients compared to AD patients

Among the 12 included articles, 8 articles have investigated the prevalence of hypertension in DLB and AD patients. 4 studies have shown that the severity of hypertension in AD patients is more or approximately equal to that of DLB patients. The prevalence of hypertension in DLB patients ranges from 21% to 56% and in AD patients from 30% to 52% (18–21). However, 4 studies had different results and showed the severity of hypertension was higher in DLB patients. The prevalence of hypertension in DLB patients ranges from 38% to 60% and in AD patients from 28% to 54% (22–25).

### The prevalence of diabetes mellitus in DLB patients compared to AD patients

Among 8 studies conducted on the prevalence of diabetes mellitus in DLB and AD patients, 3 of them show that the prevalence of diabetes is almost equal between the two groups. The prevalence of diabetes in DLB patients ranges from 9% to 31% and in AD patients from 7% to 32% (19, 21, 24). However; 3 studies show a higher prevalence of diabetes in DLB patients. The prevalence of diabetes in DLB patients ranges from 18.7% to 37% and in AD patients from 9% to 17.5% (18, 23, 25), and 2 studies show a higher prevalence of diabetes in AD patients. The prevalence of diabetes in DLB patients ranges from 4.5% to 16.7% and in AD patients from 10% to 25% (20, 22).

### Investigating the amount of WMH in different brain regions in DLB and AD patients

Among the 12 included studies, 3 reported the distribution of WMH in different cerebral lobes in patients with DLB and AD (22, 26, 27). In 7 other studies, the amount of WMH was categorized as deep white matter hyperintensities (DWMH) and periventricular white matter hyperintensities (PWMH) (18, 19, 22, 23, 26–28). Additionally, 2 studies reported the total amount of WMH in the whole brain of AD and DLB patients (19, 23).

Dadar et al.'s study showed that the amount of WMH in four brain lobes of DLB patients (parietal = 5.74 ± 5.73 cm^3^, occipital = 2.70 ±  1.93 cm^3^, frontal = 7.00 ± 4.72 cm^3^ and temporal = 2.11 ± 2.37 cm^3^) and AD patients (parietal = 3.97 ± 3.75 cm^3^, occipital = 2.01 ± 1.15 cm^3^, frontal = 6.13 ± 5.10 cm^3^ and temporal = 1.69 ± 0.93 cm^3^), which was generally higher in comparison to the control group (26). Additionally, Barber et al.'s showed a significantly higher level of WMH in DLB patients (parietal = 15 ± 1 mm, occipital = 2 ± 0 mm, frontal = 22 ± 2 mm, and temporal = 3 ± 0 mm) and AD (parietal = 13 ± 0 mm, occipital = 3 ± 0 mm, frontal = 25 ± 2 mm and temporal = 4 ± 0 mm) (27). In addition, Caso et al.'s study shows that the WMH total volume was considerably higher in DLB and AD patients in all four lobes of the brain in comparison to controls (DLB=4.6 ± 4.8 ml, AD = 1.7 ± 1.5 ml). Although WMH total volume was higher in DLB than in AD, there was no statistically significant difference between the two groups (22).

Seven studies have investigated PWMH and DWMH in AD and LBD patients (18, 19, 22, 23, 26–28). Out of seven studies, six studies showed that the amount of WMH in AD and DLB patients was non-significantly higher; Park et al. (DLB: DWMH = 1.29 ± 0.55 mm, PWMH = 1.49 ± 0.63 mm. AD: DWMH = 1.35 ± 0.67 mm, PWMH = 1.42 ± 0.60 mm) (25), Joki et al. (DLB: DSWMH = 1.74 ± 0.63, PVH = 1.92 ± 0.70. AD: DSWMH = 1.70 ± 0.61, PVH = 1.92 ± 0.72) (24), Baik et al. (DLB: DWMH = 1.5 ± 0.6 mm, PWMH = 1.8 ± 0.8 mm. AD: DWMH = 1.6 ± 0.6 mm, PWMH = 1.5 ± 0.7 mm) (28), Burton et al. (DLB: Total PVH (percent brain volume) = 0.3 (0.2–0.7) ml, Total DWMH (percent brain volume) = 0.05 (0.01–0.17) ml, AD: Total PVH (percent brain volume) = 1.0 (0.4–3.0) ml, Total DWMH (percent brain volume) = 0.24 (0.06–0.94) ml) (18), Saeed Mirza et al. (DLB: DWMH = 0.5 ± 0.8 cm^3^, PWMH = 2.5 ± 3.4 cm^3^. AD: DWMH = 0.8 ± 1.0 cm^3^, PWMH = 6.0 ± 9.3 cm^3^) (20), And a study by Bike et al. In 2023(DLB: DWMH = 1.5 ± 0.6 mm, PWMH = 1.8 ± 0.8 mm. AD: DWMH = 1.6 ± 0.6 mm, PWMH = 1.5 ± 0.7 mm) (29).

On the other hand, Sarro et al.'s study demonstrated that the amount of WMH (total WMH in DLB = 25.3 ± 21.1 cm^3^, total WMH in AD = 24.3 ± 21.9 cm^3^) was generally higher in periventricular, subcortical, and deep lobes in DLB and AD patients than in the control group. While DLB patients showed a higher amount of WMH than AD patients, after adjusting for sex and age (21).

Two studies have investigated the amount of WMH in the brain of DLB and AD patients in general (19, 23). The study of Mendes et al. (total WMH in DLB = 1.5 ± 0.92 mm, total WMH in AD = 1.7 ± 0.93 mm) (23) and Fukui et al. (total WMH in DLB = 1.05 ± 0.71, total WMH in AD = 1.00 ± 0.77) showed that the amount of WMH was significantly higher in DLB and AD patients than in the control group (19).

### Imaging sequences

The imaging sequences used in the 12 studies involving the measurement of WMH in AD and DLB patients were T2-weighted (T2W), and fluid-attenuated inversion recovery (FLAIR). Also, T-1 weighted (T1W) can be used to assist in the differential diagnosis (18–29). Seven studies have used T1w (19, 20, 23–26, 28) and eight studies have employed T2w (21, 23–29) imaging to examine intraparenchymal signal alteration in brains of AD and DLB patients. Also, five studies used FLAIR imaging (18, 19, 21, 23, 26). However, more studies have used multiple imaging modalities to assess the extent of WMH more precisely.

### Distribution and frequency of infarct in various brain regions in AD and DLB patients

In 2017, Sarro et al. conducted a study on the frequency of infarcts in cortical, subcortical, and infratentorial areas of AD and DLB patients in a control group. The results of Sarro et al.'s study show that the overall frequency of infratentorial, cortical, and subcortical infarcts was similar between the control group and DLB patients, while AD patients showed more infarct frequencies in infratentorial and cortical areas in comparison to the control group. However, AD patients had more infarcts in the cortical area than DLB patients (21). De Reuck et al.'s study examined the frequency of micro infarcts (MIs) and gross infarcts in AD and DLB patients compared to a control group. The results of this study showed that gross infarctions were present in 12.5% of DLB patients and 31% of AD patients, while no gross infarcts were observed in the control group. MIs were found in 17.5% of DLB patients and 15.6% of AD patients, compared to 10% of the control group (30).

### Sensitivity analysis

**Figure 5:**
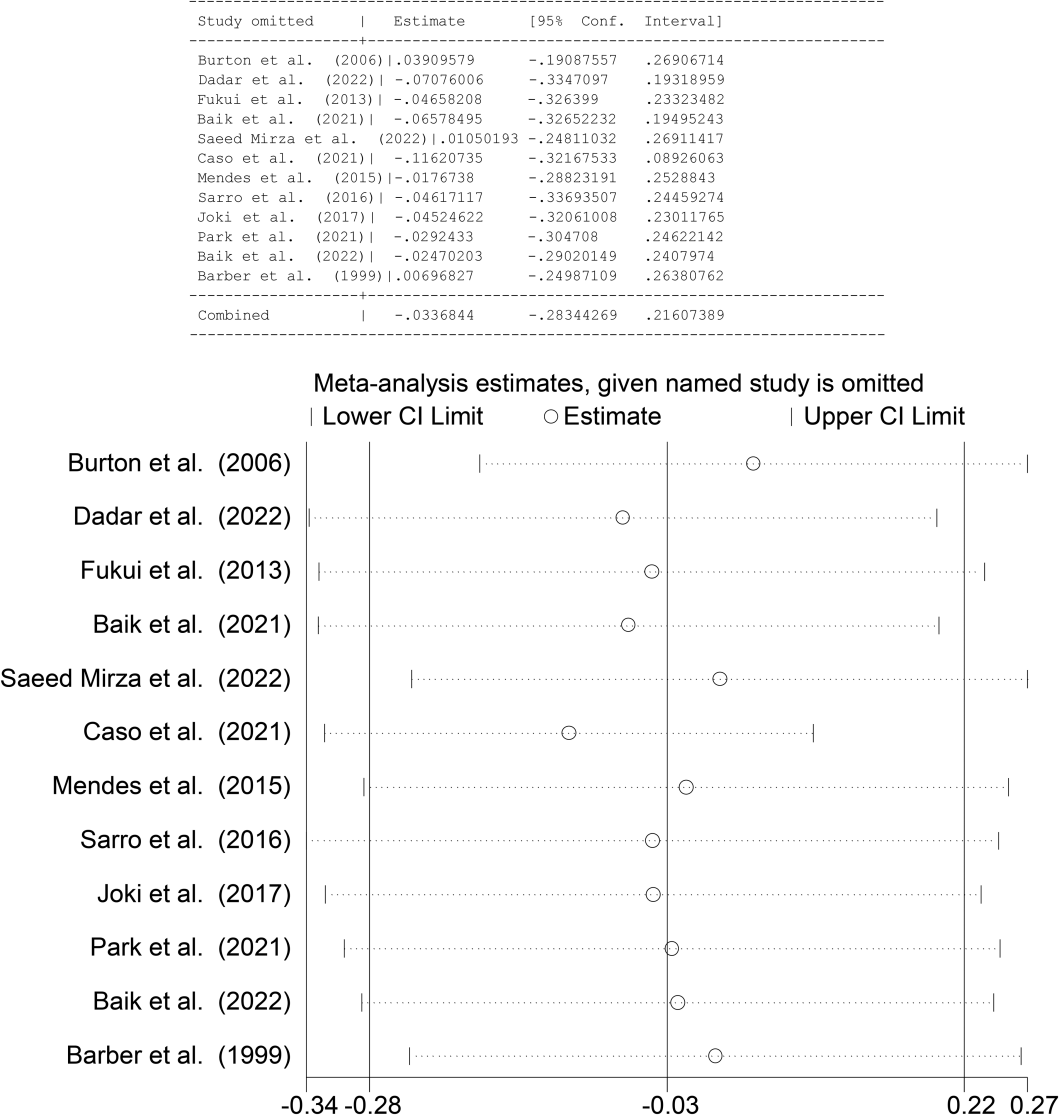
Sensitivity analysis of included studies.

The results of the sensitivity analysis demonstrated the robustness of the findings by showing that the effect size and its related 95% CI were not significantly impacted by any one research or cluster of studies with similar characteristics. Sensitivity analysis disproved the null hypothesis based on any single study or collection of studies that had statistically significant outlier features. The overall robustness of the findings was confirmed by the low impact of all studies on the 95% confidence interval and effect size (Figure 5).

### Publication bias

Figure 1 shows Begg's funnel plot based on WMH between patients with LBD and AD. The interpretation of our Begg's funnel plot (*p* = 0.681), as well as Egger's test (*p* = 0.748), demonstrates that the included studies have no publication bias. Therefore, it makes sense that studies with both positive and negative results have been released (Figures 1, 2).

### Quality assessment

We examined the quality of 12 included articles based on the quality level using the Newcastle-Ottawa Scale (NOS) (31). Among the 12 articles, 4 articles scored 9 (very good studies), 5 articles scored 8 (good studies), and 3 articles scored 7 (satisfactory studies).

## Discussion

In the present study, we investigated the amount of WMH in different brain lobes and assessed its differential role in AD and DLB. Data analysis showed that the amount of WMH in AD patients is about 0.03 ml (95% CI: −0.28–0.22; I2 = 75.4%) higher than in DLB patients. In this discussion, we seek to elucidate the differences in WMH volume between patients with AD and DLB by conducting a meta-analysis of existing studies. It is found that the neurological symptoms of patients with AD and DLB worsen as people age. They both cause a steady cognitive decline that necessitates care and assistance and place a significant psychological and socioeconomic strain on families and communities; as a result, it is imperative that physicians have the necessary training to correctly identify these disorders. As we researched into the depth of understanding AD and DLB, the diagnostic significance of WMH reveals itself as an indicator of cognitive decline. AD and DLB patients suffer from visuospatial dysfunction, impaired memory, and many similar neurological symptoms, which is why it is difficult to differentiate between these two diseases based on clinical symptoms. Nowadays, the amount of WMH in different brain lobes can be evaluated by MRI, especially using T2W methods, which is one of the differential diagnostic methods between these two diseases. Based on our analysis, the WMH volume is 0.03 ml higher in AD patients than in DLB patients, although this difference is not statistically significant. This finding contrasts with previous studies, such as those by the Sarro group, which suggested that WMH volumes were significantly higher in DLB patients compared to AD patients.

In this study, we reviewed published studies on this matter, and indicated that DLB had a lower volume of WMH than AD patients but it was not statistically significant. It is worth highlighting that studies on the relevancy of AD and WMH concluded that there are no associations between vascular risk factors such as hypertension and the risk of AD incidence (32). Nevertheless, we were unable to find definite conclusions on the relevancy of hypertension and diabetes mellitus with LBD disorders in this study due to scattered data on this matter. Several studies are conducted to use WMH as a diagnostic factor among clinical undistinguishable neurodegenerative disorders. For example, in a meta-analysis done by Liu et al. (33), they have mentioned that WMH burden is significantly higher in PDD and PD with mild cognitive impairment (PD-MCI) than patients with PD without dementia (PD-ND). Therefore, WMH can be applied as an imaging marker for PDD patients. Also, early onset AD showed greater WMH that cognitively normal and early onset amyloid negative cognitively impaired AD across all regions of brain (34).

Previous research did not provide a clear explanation for the relationship between the severity of WMH and the course of cognitive loss in AD (35). However, in DLB patients, the severity of WMH has a direct relation with cognitive decline. Another finding made by Debette et al. (36) and Prins et al. (37) is that the risk of dementia is directly correlated with the degree of the disease and change of WMH. This result is supported by a review of multiple studies by Mortamais et al. (38). They also indicated that WMH leads independently to dementia, and it is differed from the amyloid pathway. In addition, Mortmais et al. (39) clarified that severe total WMH load with a high proportion of hyper-intensities in the temporal region was significantly associated with the risk of developing MCI or dementia.

The theories on how WMH can cause cognitive impairments have been widely discussed in the literature. One theory suggests that WMH leads to cognitive decline through diminished connectivity of nerve fibers and nerve damage caused by focal hypoxia and blood-brain barrier dysfunction. This results in a disrupted neuronal network, leading to cognitive deficits. Tomimoto et al. (40) demonstrated that neuropsychological symptoms are linked to WMH or destruction of the neuronal network in the corresponding brain territory. Another explanation is that cognitive decline in patients with significant WMH, especially in the periventricular area, is associated with atrophic changes in multiple brain regions, including the hippocampus (32, 40). Studies have also shown that the severity of dementia due to WMH is influenced by factors such as cognitive reserve, WMH volume, lesion concomitancy, and their relevance to the onset of cognitive deterioration (41).

The choice of MRI sequences plays a crucial role in detecting and quantifying WMH for differentiating AD from DLB. Seven studies used T1W sequences in our analysis, whereas eight used T2W, and FLAIR was incorporated in five. T1W images tend to underestimate WMH load because they are not as sensitive to white matter lesions, whereas T2W provides better lesion contrast yet fails to suppress CSF, which is not helpful when assessing periventricular lesions (36). FLAIR is considered best sensitive for the detection of WMH since it enhances contrast by suppressing CSF signals for accurate volume estimation (32).

With these modality-dependent differences, some variation in reported WMH burden across studies may be ascribed to differences in imaging protocols. The limited application of FLAIR in some studies may also raise issues of WMH underestimation, possibly contributing to prejudices in disease characterization. The comparability of findings can be enhanced by standardizing multimodal imaging approaches whereby FLAIR would combine with T2W and T1W sequences to improve the reliability of WMH evaluation and facilitate cross-study comparability (14). Further studies should also delve into how different modality selection may influence clinical correlations, more importantly, cognitive decline and disease progression in AD and DLB. A common effort in addressing these methodological discrepancies is crucial in future developmental work towards refining imaging-based biomarkers to inform diagnostic accuracy in neurodegenerative research. The volume of WMH is also important, as some patients with high WMH volumes do not experience cognitive decline, while others with slight increases in WMH volume may suffer severe cognitive impairment (42). The location of WMH is significant too; temporal WMH, for example, is linked to memory problems (43). Lesion-symptom mapping studies have found poor memory associated with lesions in temporo-occipital white matter, the internal capsule, and the corpus callosum, although the evidence is not conclusive (42). Comparing AD and DLB, it was found that AD patients had less WMH volume in the posterior periventricular and occipital regions than DLB patients. However, this remains a complex issue requiring further research.

Collecting data on the causes, consequences, and co-occurring brain disorders that contribute to dementia is vital for improving diagnosis and treatment, thereby reducing the social burden on patients, caregivers, and healthcare systems. Subsequently, in order to lessen the burden or extend the patient's useful life, these progressive illnesses should be given a lot of attention to identify novel diagnostic features or investigate concurrent implications that lead to cognitive decline. Therefore, more research on this matter and the way to manage or treat this condition are required. This paper suggests that research on the targeted interventions in WMH may help the patients to have a better quality of life, help their caregivers to decrease their stressful lives, and also help the government by reducing the burden of patients with dementia in hospitals and society.

## Limitation

A limitation of this research was that certain studies that included LBD illnesses did not show that the findings applied to people with DLB. Papers written in languages other than English were also excluded. It is also crucial to remember that there is a possibility of publication bias in this area because the incidence of WMH can also be caused by a number of extremely common conditions around the world, such as DM or HTN, in addition to AD and DLB. Therefore, researches on patients with no previous disorders may be lightening and help the generalizability of the results.

## Conclusion

This study underscores the importance of WMH as a potential diagnostic marker for differentiating between AD and dementia with Lewy bodies (DLB). While our meta-analysis indicates that AD patients have a slightly higher WMH volume than DLB patients, the difference is not statistically significant. This finding suggests that while WMH can contribute to the diagnostic process, it should be used in conjunction with other diagnostic modalities. Further research is essential to understand the underlying mechanisms, improve diagnostic accuracy, and explore therapeutic strategies to manage WMH-related cognitive decline. Addressing these issues will enhance patient care, alleviate the burden on caregivers, and reduce the societal impact of these neurodegenerative diseases.

## Data Availability

The original contributions presented in the study are included in the article/Supplementary Material, further inquiries can be directed to the corresponding authors.
